# Synergistic effect of immune checkpoint blockade and anti-angiogenesis in cancer treatment

**DOI:** 10.1186/s12943-019-0974-6

**Published:** 2019-03-30

**Authors:** Ming Yi, Dechao Jiao, Shuang Qin, Qian Chu, Kongming Wu, Anping Li

**Affiliations:** 1 Department of Oncology, Tongji Hospital of Tongji Medical College, Huazhong University of Science and Technology, Wuhan, 430030 China; 2 Department of Interventional Radiology, The First Affiliated Hospital of Zhengzhou University, Zhengzhou, 450052 China

**Keywords:** Immune checkpoint inhibitor, PD-1, PD-L1, CTLA-4, VEGF, Anti-angiogenesis, TKI, Tumor immune microenvironment

## Abstract

Immune checkpoint inhibitor (ICI) activates host’s anti-tumor immune response by blocking negative regulatory immune signals. A series of clinical trials showed that ICI could effectively induce tumor regression in a subset of advanced cancer patients. In clinical practice, a main concerning for choosing ICI is the low response rate. Even though multiple predictive biomarkers such as PD-L1 expression, mismatch-repair deficiency, and status of tumor infiltrating lymphocytes have been adopted for patient selection, frequent resistance to ICI monotherapy has not been completely resolved. However, some recent studies indicated that ICI resistance could be alleviated by combination therapy with anti-angiogenesis treatment. Actually, anti-angiogenesis therapy not only prunes blood vessel which is essential to cancer growth and metastasis, but also reprograms the tumor immune microenvironment. Preclinical studies demonstrated that the efficacy of combination therapy of ICI and anti-angiogenesis was superior to monotherapy. In mice model, combination therapy could effectively increase the ratio of anti-tumor/pro-tumor immune cell and decrease the expression of multiple immune checkpoints more than PD-1. Based on exciting results from preclinical studies, many clinical trials were deployed to investigate the synergistic effect of the combination therapy and acquired promising outcome. This review summarized the latest understanding of ICI combined anti-angiogenesis therapy and highlighted the advances of relevant clinical trials.

## Background

Immune checkpoint molecules mainly includes programmed cell death protein 1 (PD-1) and cytotoxic T-lymphocyte antigen-4 (CTLA-4) [1–4]. As the vital components of immune homeostasis, immune checkpoint molecules downregulate magnitude of immune response and participate in peripheral tolerance [5]. However, upregulated immune checkpoint signaling pathways such as PD-1/PD-L1 protect cancer cell from immune surveillance [6]. Therefore, immune checkpoint molecules and their ligands are ideal anti-cancer treatment targets. It is well established that anti-PD-1/PD-L1 upregulates Ras-Raf-MEK-ERK and PI3K-AKT signaling pathways in immune cells by blocking PD-1/PD-L1 axis [7]. As a result, anti-PD-1/PD-L1 therapy restores T cell from exhausted status and enhances tumor-killing activity [8]. Relatively, mechanisms by which anti-CTLA-4 therapy destroys cancer cell are still controversial. It is generally believed that anti-CTLA-4 recovers the co-stimulatory signaling pathway CD28-B7 which is usually hijacked by CTLA-4 in tumor microenvironment [9, 10]. Additionally, it is proposed that anti-CTLA-4 could directly eliminate regulatory T (Treg) cell by antibody-dependent cell-mediated cytotoxicity [11–13].

Compared with immune checkpoint inhibitor (ICI), anti-angiogenesis therapy attracted intensive attention earlier. Angiogenesis, mainly indicating the generation of new vessels from pre-existing ones, occurs in many physiological processes (e.g. wound healing) [14]. In the meanwhile, angiogenesis participates in the growth and metastasis of solid tumor [15]. Due to the characteristics of the rapid division and growth, tumor cell consumes a large amount of oxygen and nutrients. Besides, active metabolism with disproportional blood supply leads to hypoxia and acidosis in tumor bed [15, 16]. Subsequently, hypoxia induces tumor and stroma cells to secret multiple pro-angiogenic factors such as vascular endothelial growth factor (VEGF), basic fibroblast growth factor (bFGF), and matrix metalloproteinase (MMP) [17]. As a result, the local balance of pro-angiogenic factors and anti-angiogenic factors is disturbed and multiple angiogenic pathways are activated [18]. However, due to the persistent hypersecretion of pro-angiogenic factors in tumor microenvironment, vessel maturation process is impeded [19]. Abnormal angiogenesis leads to the lack of pericyte coverage and leaky nascent vessels [20, 21]. Disorganized and leaky vessels result in increased vascular permeability and interstitial fluid pressure [22].

The initial aim of anti-angiogenesis therapy is to reduce blood supply and starve tumor cell of oxygen and nutrients [23]. However, no significant improvements in outcomes were observed in patients undergoing anti-angiogenesis therapy alone. Vessel normalization theory provides a novel perspective in anti-angiogenesis and indicates potential synergistic effect in combination with other therapies. This review focused on the application of ICI combined with anti-angiogenesis therapy.

## The influence of angiogenesis on ICI therapy

### The status of tumor infiltrating lymphocytes determines the efficacy of ICI

Tumor infiltrating lymphocyte (TIL) is one of the most important components for tumor-killing activity. For ICI therapy especially anti-PD-1/PD-L1 intervention, pre-existing TIL is the necessary precondition for potent tumor regression. Based on the status of pre-existing TIL, tumor microenvironments are classified into three types: (I) immune inflamed type, where dense functional CD8^+^ T cells infiltrate; (II) excluded infiltration type, where abnormal angiogenesis and immunosuppressive reactive stroma prevent the infiltration of T cell; (III) immune ignorance type, where tumor mutation burden and the expression of antigen presentation machinery marker are low [24]. It was verified that the tumors belonging to immune inflamed type were more sensitive to ICI therapy than two other types [25]. Moreover, treatment enhancing T cell infiltration could promote the effect of ICI [26, 27].

### Angiogenesis affects the status of TIL

**Fig. 1:**
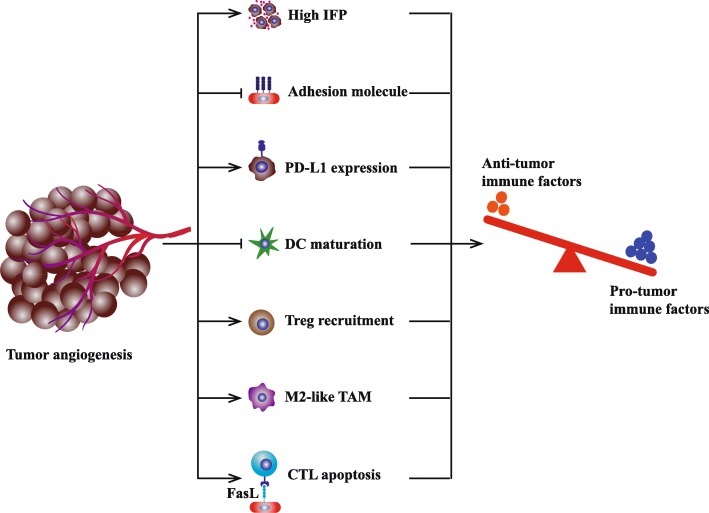
Tumor angiogenesis induces the formation of immunosuppressive tumor microenvironment. Firstly, leaky nascent vessels and loose pericyte coverage result in high interstitial fluid pressure (IFP) which means greater pressure difference to overcome for T cell infiltration. Secondly, neo-vasculatures tend to lack some adhesion molecules for example vasculature cell adhesion molecule-1 (VCAM-1). Thirdly, hypoxia upregulates some inhibitory signals for anti-tumor immune response such as PD-L1, indoleamine 2, 3-dioxygenase (IDO), interleukin-6 (IL-6), and interleukin-10 (IL-10) . In addition, circulating VEGF impedes the maturation and function of dendritic cell (DC). Besides, tumor hypoxia induces upregulation of chemokine (C-C motif) ligand-22 and chemokine (C-C motif) ligand-28, which recruit Treg into tumor [36, 37]. Moreover, hypoxic tumor microenvironment promotes the polarization of tumor-associated macrophage (TAM) to M2-like phenotype. Lastly, the expression of Fas ligand (FasL) on tumor endothelial barrier selectively eliminates effector CD8^+^ T cells rather than Treg, due to the high expression of cellular FLICE-inhibitory protein (c-FLIP) expression on Treg. In summary, angiogenesis render accumulating pro-tumor immune cells and decreasing anti-tumor immune cells, inducing the formation of immunosuppressive tumor microenvironment

In cancer-immunity cycle, the presentation of neoantigen determines the generation of tumor specific T cell clones. Then, T cells with specific T cell receptor (TCR) traffic to and infiltrate into tumor. TIL recognizes neoantigen and kills tumor cell in immunosupportive tumor microenvironment [28, 29]. For most growing solid tumors, hyperactive angiogenesis contributes to immunosuppressive microenvironment by affecting multiple immune steps (Fig. 1) [30, 31].

On the one hand, abnormal angiogenesis decreases the abundance and function of anti-tumor lymphocytes. Firstly, leaky nascent vessels and loose pericyte coverage result in high interstitial fluid pressure which means greater pressure difference to overcome for T cell infiltration. Rare T cell could cross physical barrier and infiltrates into tumor bed [32]. Secondly, neo-vasculatures tend to lack some adhesion molecules for example vasculature cell adhesion molecule-1 (VCAM-1). Downregulated adhesion molecules further impair the extravasation of T cell [32]. Thirdly, neo-vasculatures could not compensate for increased oxygen consumption and concurrent hypoxia directly undermine the functions of TIL. Hypoxia upregulates some inhibitory signals for anti-tumor immune response such as PD-L1, indoleamine 2, 3-dioxygenase (IDO), interleukin-6 (IL-6), and interleukin-10 (IL-10) [14, 33]. In addition, circulating VEGF impedes the maturation and function of dendritic cell (DC) to help tumor escape immune surveillance [34, 35].

On the other hand, hyperactive angiogenesis increases the abundance of pro-tumor lymphocytes. As the consequence of abnormal tumor vessel, tumor hypoxia induces upregulation of chemokine (C-C motif) ligand-22 and chemokine (C-C motif) ligand-28, which recruit Treg into tumor [36, 37]. Besides, hypoxic tumor microenvironment promotes the polarization of tumor-associated macrophage (TAM) to M2-like phenotype [38]. Thirdly, the expression of Fas ligand (FasL) on tumor endothelial barrier selectively eliminates effector CD8^+^ T cells rather than Treg, due to the high expression of cellular FLICE-inhibitory protein (c-FLIP) expression on Treg [39]. In summary, angiogenesis participates in tumor growth and immune evasion by multiple manners.

## Anti-angiogenesis agents: The natural ally of ICI

### Main anti-angiogenesis agents

**Fig. 2:**
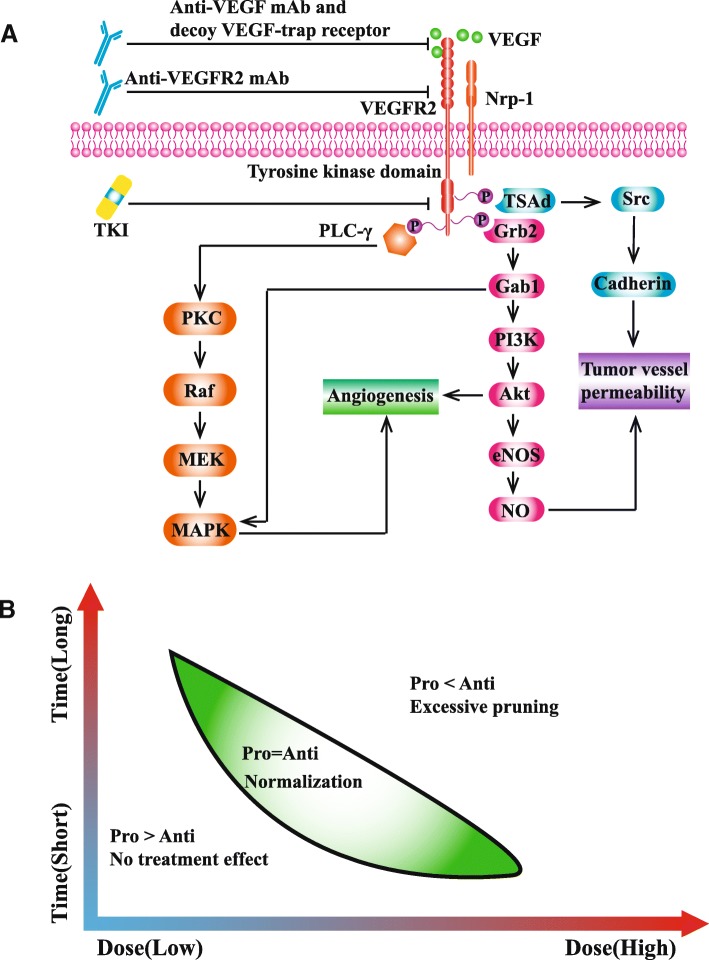
**a** Main angiogenesis pathways and anti-angiogenesis agents. VEGF-VEGFR2 promotes the proliferation and migration of endothelial cell primarily by activating downstream PLCγ-PKC-Raf-MAPK and Grb2-Gab1-MAPK/PI3K-Akt signaling pathways. In addition, VEGF-VEGFR2 could increase vascular permeability by activating VEGFR2–TSAd–Src-cadherin and PI3K–Akt–eNOS–NO signaling pathways. Anti-angiogenesis agents consist of three types: (I) anti-VEGF monoclonal antibody (mAb) such as bevacizumab and decoy VEGF-trap receptor such as aflibercept; (II) anti-VEGFR2 mAb (ramucirumab); (III) VEGFR tyrosine kinase inhibitor (TKI). **b** Normalization window of anti-angiogenesis treatment. When pro-angiogenic (pro) factors balance with anti-angiogenic (anti) factors, abnormal tumor vessels transform into normal-like phenotype (green). Vessel normalization is a transient status changing along with the time and dose of treatment

Solid tumors tend to secret multiple pro-angiogenetic factors such as VEGF (also known as VEGF-A), hepatocyte growth factor, and platelet derived growth factor. Among these factors, VEGF plays a core role in angiogenesis [21, 40]. The angiogenetic signal of VEGF is mainly transducted by its receptor VEGFR2 [41, 42]. VEGFR2 contains a ligand-binding domain with 7 immunoglobulin-like structures, a trans-membrane domain, and a tyrosine kinase domain [43]. On the one hand, VEGF-VEGFR2 promotes secretion of von Willebrand factor (vWF), proliferation and migration of endothelial cell (EC) by activating downstream PLCγ-PKC-Raf-MAPK and Grb2-Gab1-MAPK/PI3K-Akt signaling pathways [44]. On the other hand, VEGF-VEGFR2 could increase vascular permeability by activating VEGFR2–TSAd–Src-cadherin and PI3K–Akt–eNOS–NO signaling pathways (Fig. 2a) [23, 44]. Therefore, VEGF and its receptor VEGFR2 are predominant targets for the development of anti-angiogenesis agents. Anti-VEGF monoclonal antibody (mAb) bevacizumab is the first anti-angiogenesis agent which is approved for multiple cancers including metastatic colorectal cancer, metastatic non-squamous non-small cell lung cancer, metastatic renal cell carcinoma, recurrent glioblastoma, recurrent ovarian cancer, recurrent/metastatic cervical cancer [45]. Following the invention of bevacizumab, a variety of VEGF-VEGFR targeted agents come out. Apart from anti-VEGF mAb, there are other three approaches to inhibit VEGF-VEGFR signaling pathway: (I) decoy VEGF-trap receptor such as aflibercept [46]; (II) anti-VEGFR2 mAb such as ramucirumab [47]; (III) tyrosine kinase inhibitor (TKI) which interferes intracellular signal transduction of VEGF such as axitinib, sorafenib, sunitinib, and vatalanib [48–51]. Moreover, based on chimeric antigen receptor (CAR) T cell technology, Chinnasamy et al. developed anti-VEGFR2 CAR T cell to retard tumor growth [52]. Anti-VEGFR2 CAR-T therapy is verified as an effective strategy inducing tumor regression but its effect needs further investigation in human.

### Anti-angiogenesis: From tumor starvation to vessel normalization

For most species, the formation of functional vessel needs maturation process [19]. In the absence of VEGF, nascent vessels undergo a series of modification procedures including basement membrane deposition, EC-EC junction formation, and pericyte coverage [19]. Driven by the persistent hypersecretion of VEGF, tumor vessels do not possess tight EC-EC conjunction, sufficient pericyte coverage, and lack intact basement membrane [53, 54]. The survival of these vessel is highly dependent on activated VEGF-VEGFR2 signaling pathway [55].

Originally, anti-angiogenesis agents were developed to interfere neo-vascularization and starve tumor, but they did not yield satisfactory effect [53]. Presumably under selective pressure, tumor with excessively pruned blood vessel are prone to transform to the phenotype tolerable to hypoxia, rendering increased invasiveness and metastasis ability [56, 57]. In spite of the unsatisfactory efficacy of monotherapy, it was found anti-angiogenesis could be used as a sensitizer in combination with other therapies [58, 59]. However, there is a paradox that the elimination of tumor vessel simultaneously restrains the delivery of drug and oxygen [53]. Jain established a model to describe the transient status of tumor vessel undergoing anti-angiogenesis: vessel normalization [53]. In the model, when pro-angiogenic factors balance with anti-angiogenic factors, abnormal tumor vessels transform into a normal-like phenotype with characteristics including increased perfusion, pericyte coverage, and decreased hypoxia [53, 60, 61]. Notably, vessel normalization status depends on the schedule and dose of treatment (Fig. 2b). Huang et al. conducted a study to investigate the relationship between anti-angiogenesis dose and efficacy. The results demonstrated that lower dose of anti-angiogenesis agent was superior to higher dose treatment in inducing homogeneous tumor vessel normalization [62]. We proposed that higher dose anti-angiogenesis might result in rapider vessel pruning and shorter normalization window.

### Anti-angiogenesis: Reprograming tumor immune microenvironment

A growing body of evidence demonstrated that appropriate anti-angiogenesis administration could convert tumor immune environment from immunosuppressive to immunosupportive status [63, 64]. Normalized tumor vascular network could directly alleviate hypoxia and promote T cell infiltration. Alleviated hypoxia preferentially induces polarization of TAM to M1-like phenotype [62]. Besides, vessel normalization decreases the recruitment of Treg and myeloid-derived suppressor cell (MDSC) [14, 65]. In addition, anti-VEGF agents block the inhibitory signal for DC differentiation and decrease overall MDSC pool [66]. Lastly, hypoxia-induced inhibitory immune signals such as PD-L1 could be downregulated by improved perfusion [67].

## ICI plus anti-angiogenesis therapy in preclinical studies

**Fig. 3:**
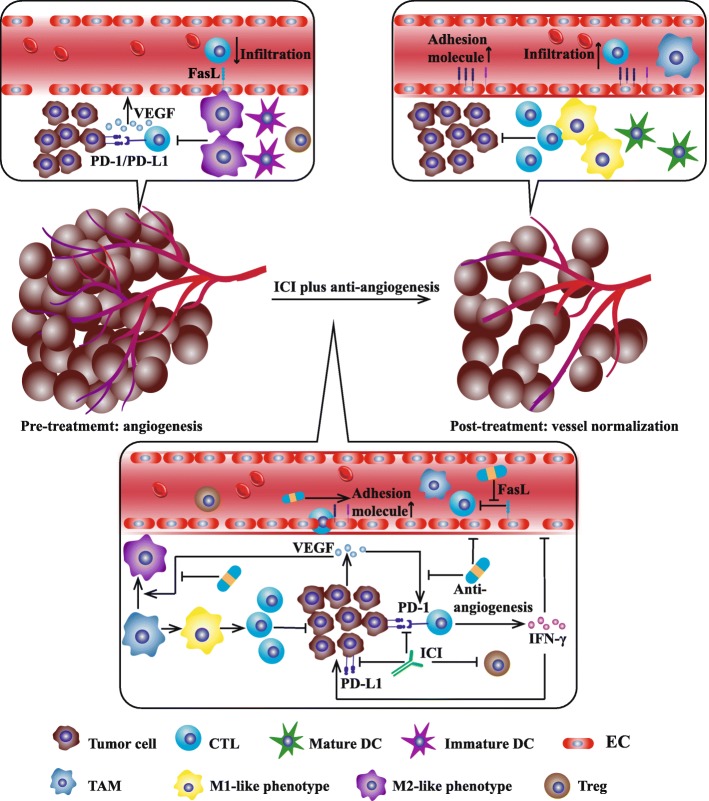
Mutual regulation of tumor vessel normalization and immune microenvironment reprogramming. Tumor angiogenesis leads to an immunosuppressive microenvironment by decreasing the ratio of anti-tumor/pro-tumor immune cell and undermining the function of cytotoxic T lymphocyte (CTL). Anti-angiogenesis induces tumor vessel normalization and improves blood perfusion. Alleviated hypoxia decreases PD-L1 expression on tumor cell while blocked VEGF signal downregulates immune checkpoint expression (e.g. PD-1) on CTL. In the meanwhile, activated immune response-derived inflammatory factors such as interferon-γ (IFN-γ) promotes vessel normalization and regression. Interaction between vessel normalization and immune microenvironment reprogramming could be regulated by anti-angiogenesis agents (bevacizumab or VEGFR-TKI such as axitinib, sorafenib, sunitinib, and vatalanib) and ICI (especially anti-PD-1/PD-L1 mAb). After combination therapy, immunosuppressive microenvironment is transformed to immunosupportive microenvironment which possesses increased CTL, M1-like phonotype macrophage, adhesion molecule, mature dendritic cell (DC), and decreased regulatory T cell (Treg). Abbreviations: TAM, tumor associated macrophage; EC, endothelial cell

Tumor immune escape closely relates to angiogenesis. In turn, tumor angiogenesis highly depends on immunosuppressive microenvironment. Activated T cell secrets interferon-γ (IFN-γ) which could directly promote tumor vessel normalization and regression by IFN-γ receptor on tumor endothelial cell (Fig. 3) [68–70]. Based on the interaction between tumor immunity and angiogenesis, it is speculated that anti-angiogenesis might enhance the efficacy of ICI. As early as 2013, Yasuda et al. observed the synergistic effect between ICI and anti-angiogenesis in mice bearing colon adenocarcinoma [71]. Subsequently, Wu et al. verified that ICI plus anti-angiogenesis could effectively prolong overall survival (OS) in mice bearing kidney and mammary tumors [72]. However, apart from decreased interstitial fluid pressure and correspondingly improved T cell infiltration, we could not rule out other mechanisms by which ICI and anti-angiogenesis synergistically kill tumor cell. Thus, further explorations should be conducted in expanding models. To date, multiple mechanisms have been found to relate to synergistic effect.

### Blocking VEGF-induced immune checkpoint expression

Meder et al. conducted a preclinical study by genetically engineered small-cell lung cancer (SCLC) mouse [73]. All SCLC-bearing mice were randomly assigned into five groups and received the following therapies: (I) phosphate buffered saline (vehicle); (II) IgG; (III) anti-VEGF mAb (B20–4.1.1-PHAGE); (IV) anti-PD-L1 mAb (clone 6E11); (V) anti-VEGF plus anti-PD-L1 [73]. Among 5 groups, combination therapy group possessed the best survival data [73]. Moreover, compared with mice sensitive to anti-PD-L1, the abundance of exhausted T cell (PD-1^+^/TIM-3^+^/LAG-3^+^ T cell) significantly increased in mice resistant to anti-PD-L1 [73]. However, the increased ratio of exhausted T cells could be reversed by following anti-VEGF plus anti-PD-L1 treatment [73]. To confirm the influence of VEGF on immune checkpoint expression, human T cell was obtained from peripheral blood of SCLC patients [73]. After stimulation with VEGF, the expression of PD-1 and TIM-3 on T cell was significantly upregulated [73].

In line with the finding of Meder and colleagues, Voron et al. observed that anti-VEGF could selectively inhibit the expression of immune checkpoint molecules (e.g. PD-1, CTLA-4, and TIM-3) on intratumoral CD8^+^ T cell [74]. Voron et al. found that VEGF could upregulate the expression of PD-1 by activating VEGFR2-PLCγ-calcineurin-NFAT signaling pathway [74]. Therefore, anti-PD-1 therapy together with anti-VEGF could effectively block PD-1/PD-L1 axis and synergistically suppress tumor growth, especially for tumor with VEGF hypersecretion [74].

### Impairing IFN-γ-mediated negative feedback

Apart from VEGF signaling pathway, angiopoietin-2 (ANGPT2)/Tie 2 is another pro-angiogenic pathway which relates with resistance to anti-VEGF treatment [75–77]. Schmittnaegel et al. confirmed that the dual blockade of VEGF and ANGPT2 by bispecific antibody A2V provided a more potent therapeutic effect than monotherapy [78]. In the meanwhile, the treatment effect of dual blockade could be further enhanced by anti-PD-1 treatment [78]. In this preclinical study, multiple tumor bearing mouse models were employed including transgenic or transplanted breast cancer, pancreatic neuroendocrine cancer, melanoma, and colorectal adenocarcinoma models [78]. After A2V treatment, the abundance of anti-tumor immune cells including mature DC, M1-like phenotype TAM, IFN-γ^+^/CD69^+^ CD8^+^ T cell increased [78]. In the meanwhile, increased perivascular CD8^+^ T cells accompanied the high expression of PD-L1 on tumor cell because of IFN-γ-mediated negative feedback regulatory mechanism [78]. Combination therapy of anti-PD-1 and A2V blocked the negative feedback loop and magnified the immune response [78]. The results showed that more than 30% mice receiving combination therapy possessed prolonged OS compared with A2V therapy [78].

### Inducing high endothelial venule formation

Allen et al. investigated the efficacy of combination therapy of anti-PD-L1 (anti-PD-L1 mAb: B20S) and anti-VEGFR2 (anti-VEGFR2 mAb: DC101) in mice bearing pancreatic neuroendocrine tumor, mammary carcinoma, and glioblastoma [79]. Combination therapy showed a great advantage in tumor control and OS over monotherapy in pancreatic neuroendocrine tumor and mammary carcinoma but for glioblastoma [79]. After 2 weeks treatment of anti-PD-L1 plus anti-VEGFR2, the level of IFN-γ^+^ CD8^+^ and IFN-γ^+^ CD4^+^ T cell increased by twofold in pancreatic neuroendocrine tumor and mammary carcinoma. However, IFN-γ^+^ CD8^+^ T cell modestly increased in just 50% of glioblastomas [79]. As the direct barrier for T cell extravasation, intratumoral vessel was speculated as the primary factor contributing to the impeded T cell infiltration in glioblastomas [79]. Apart from more intact pericyte coverage, vessel in pancreatic neuroendocrine tumor and mammary carcinoma was thickened with plump endothelial cells rather than flat endothelial cells, displaying the unique characteristic of high endothelial venule (HEV) [79]. Immunohistochemical analysis confirmed this phenotype transformation of endothelial cell. It is generally believed that HEV is associated with lymphocyte homing [80–82]. Similarly, it was speculated that intratumoral HEV promoted T cell infiltration into tumor [83]. LTβR signaling pathway is essential to sustain HEV phenotype [79]. Activating LTβR signaling pathway by its agonist during combination therapy could effectively eliminate glioblastoma, indicating the vital role of HEV formation in combination therapy [79].

## ICI plus anti-angiogenesis therapy in clinical studies

As discussed above, the interaction between immunity and angiogenesis renders tumor immune escape and treatment resistance. Based on the encouraging results of preclinical studies, many clinical studies have been conducted to investigate the synergistic effect of ICI plus anti-angiogenesis therapy in patients (Table 1). Schmidt et al. established a mathematical model to evaluate synergistic effect of multiple anti-PD-1-based combination therapies including anti-PD-1 plus chemotherapy, anti-angiogenesis, or anti-CTLA-4 treatment [84]. By subtracting the independent contributions of combination therapies from overall treatment effect, it was calculated that anti-PD-1 plus anti-angiogenesis therapy possessed the strongest synergistic effect among all combination strategies [84].

**Table 1:** Clinical trials investigating the efficacy of ICI plus anti-angiogenesis therapy

Trials Identifier	Disease	Treatment (arm of combination therapy)	Phase	Status
NCT03024437	RCC	Atezolizumab + bevacizumab + entinostat	I/II	Recruiting
NCT03363867	OC	Atezolizumab + bevacizumab + cobimetinib	II	Recruiting
NCT03472560	NSCLC/UC	Avelumab + axitinib	II	Recruiting
NCT03395899	BC	Atezolizumab + bevacizumab + cobimetinib	II	Recruiting
NCT02724878	NCCKC	Atezolizumab + bevacizumab	II	Recruiting
NCT03386929	NSCLC	Avelumab + axitinib + palbociclib	I/II	Recruiting
NCT03574779	OC	TSR-042+ bevacizumab + Niraparib	II	Recruiting
NCT02921269	CC	Atezolizumab + bevacizumab	II	Active, not recruiting
NCT03647956	NSCLC	Atezolizumab + bevacizumab + carboplatin + pemetrexed	II	Recruiting
NCT02734004	OC/BC/SCLC/GC	MEDI4736 + bevacizumab + olaparib	I/II	Recruiting
NCT03517449	EC	Pembrolizumab + lenvatinib	III	Recruiting
NCT02572687	GC/GEJ/NSCLC/HCC	MEDI4736 + ramucirumab	I	Active, not recruiting
NCT02839707	OC/FTC/PC	Atezolizumab + bevacizumab + PLD	II/III	Recruiting
NCT03289533	HCC	Avelumab + axitinib	I	Recruiting
NCT02210117	RCC	Ipilimumab + bevacizumab	I	Active, not recruiting
NCT01950390	Melanoma	Ipilimumab + bevacizumab	II	Active, not recruiting
NCT03394287	BC	SHR-1210 + apatinib	II	Recruiting
NCT03417895	SCLC	SHR-1210 + apatinib	II	Not yet recruiting
NCT03491631	Multiple solid tumors	SHR-1210 + apatinib + SHR9146	I	Not yet recruiting
NCT02942329	HCC/GC	SHR-1210 + apatinib	I/II	Recruiting
NCT03671265	ESCC	SHR-1210 + apatinib + radiation	NA	Not yet recruiting
NCT03359018	Osteosarcoma	SHR-1210 + apatinib	II	Active, not recruiting
NCT03722875	HCC	SHR-1210 + apatinib	NA	Not yet recruiting
NCT03502746	Mesothelioma	Nivolumab + ramucirumab	II	Recruiting
NCT03606174	UC	Nivolumab + sitravatinib	II	Recruiting
NCT02853331	RCC	Pembrolizumab + axitinib	III	Active, not recruiting
NCT03680521	RCC	Nivolumab + sitravatinib	II	Recruiting
NCT02493751	RCC	Avelumab + axitinib	I	Active, not recruiting
NCT02684006	RCC	Avelumab + axitinib	III	Active, not recruiting
NCT02366143	NSCLC	Atezolizumab + bevacizumab + paclitaxel + carboplatin	III	Active, not recruiting
NCT00790010	Melanoma	Ipilimumab + bevacizumab	I	Active, not recruiting
NCT 01633970	Multiple solid tumors	Atezolizumab + bevacizumab	I	Active, not recruiting

The details of Table 1 was obtained from http://clinicaltrials.gov/. Abbreviations: *BC* breast cancer, *CC* cervical cancer, *EC* endometrial cancer, *ESCC* esophageal squamous cell carcinoma, *FTC* fallopian tube cancer, *GC* gastric cancer, *GEJ* gastroesophageal junction adenocarcinoma, *GIST* gastrointestinal stromal tumor, *HCC* hepatocellular carcinoma, *NA* not applicable, *NCCKC* Non-clear cell kidney cancer, *NSCLC* non-small cell lung cancer, *OC* ovarian cancer, *PC* peritoneal cancer, *PLD* pegylated liposomal doxorubicin hydrochloride, *RCC* renal cell cancer, *SCLC* small cell lung cancer, *UC* urothelial cancer

### Anti-CTLA-4 combined with anti-VEGF mAb

NCT00790010 is a phase I clinical trial to explore the effect of ipilimumab (anti-CTLA-4) plus bevacizumab (anti-VEGF) in metastatic melanoma patients [85]. All 46 recruited patients were classified into 4 cohorts and received different dosages of combination therapy [85]. It was observed that combination therapy significantly promoted upregulation of CD31, E-selectin, VCAM-1, and other adhesion molecules on intratumoral endothelia cell [85, 86]. In the same time, trafficking of cytotoxic T cell and mature DC were enhanced [85]. Compared with the results of previous studies, patients undergoing combination therapy showed a great advantage in prognosis (median OS, ipilimumab plus bevacizumab vs. ipilimumab: 25.1 vs. 10.1 months) [85, 87]. Further exploration revealed that the favorable effect of combination therapy might derive from induced immune response to galectin-1 (Gal-1) [88]. Gal-1 is a versatile molecule participating in proliferation, invasion, immune escape, and angiogenesis processes [89, 90]. Patients’ plasma samples were collected to detect the titer of anti-Gal-1 antibody. The results showed that 62.5% of complete response/partial response patients had increased anti-Gal-1 antibody titer (≥ 1.5 fold), while just 36.4% of stable disease patients and 23.1% of progressive disease patients had increase in anti-Gal-1 antibody titer after treatment [89]. Different responses to combination therapy were attributed to distinct anti-Gal-1 immune responses [88]. It was proposed that two factors leaded to the emergency of anti-Gal-1 antibody. On the one hand, anti-VEGF could upregulate the generation of Gal-1 [91]. On the other hand, anti-CTLA-4 increases the phenotypes of T cell clones. The two factors elevate the probability of Gal-1 recognition by antigen presentation cell [88]. In addition, two other clinical trials (NCT02210117 and NCT01950390) investigating the effect of combination therapy of ipilimumab plus bevacizumab are ongoing. These two clinical trials involved metastatic kidney cancer and stage III-IV melanoma patient respectively.

### Anti-PD-L1 combined with anti-VEGF mAb

Inspired by the significantly synergistic effect of anti-CTLA-4 plus anti-VEGF therapy, Wallin et al. conducted the clinical study (NCT 01633970) to explore the efficacy of anti-PD-L1 combined with anti-VEGF [26]. NCT01633970 is a phase 1b study aiming to investigate the safety and pharmacology of atezolizumab plus bevacizumab or chemotherapy [26]. 10 metastatic renal cell cancer patients received 1 cycle bevacizumab monotherapy followed by combination therapy until disease progression or unacceptable adverse event [26]. 8 of 10 patients showed partial response or stable disease [26]. The results of this small cohort were significantly better than previous monotherapy studies [92, 93]. Compared with tumor samples from patients at baseline or post bevacizumab monotherapy, the expression of CD8, PD-L1, and major histocompatibility complex-I (MHC-I) markedly increased after combination therapy [26]. The transformation to hot tumor was associated with increased expression of CX3CL1 which participated in the recruitment of peripheral CD8^+^ T cells [26]. Dynamic TCR sequencing analysis demonstrated evolving TCR repertoire during treatment [26]. The emergency of new clones relates to trafficking of tumor specific T cell and contributes to tumor control [26].

In 2018, the results of the phase 3 study IMpower150 (NCT02366143) were reported. This study was aimed to evaluate the effect of combination therapy consisting of atezolizumab, bevacizumab, and chemotherapy in treatment-naïve metastatic non-squamous non–small-cell lung cancer patients [94]. Among total 2166 enrolled patients, 400 patients received atezolizumab plus bevacizumab plus carboplatin plus paclitaxel therapy (ABCP group) while other 400 patients received bevacizumab plus carboplatin plus paclitaxel therapy (BCP group) [94]. Objective response rate (ORR) of ABCP group was significantly higher than BCP group (ORR: 63.5% vs. 48.0, 95%CI: 58.2–68.5% vs. 42.5–53.6%), while adverse event rate was comparable (overall adverse event rate: 94.4% vs. 95.4%; grade 1–2 adverse event rate: 35.9% vs. 45.4%; grade 3–4 adverse event rate: 55.7% vs. 47.7%) [94]. Besides, the results of Kaplan–Meier analysis showed that both progression-free survival (PFS) and OS were significantly prolonged in ABCP group (median PFS of ABCP vs. BCP: 8.3 vs. 6.8 months; hazard ratio: 0.61, 95% CI: 0.52 to 0.72) (median OS of ABCP vs. BCP: 19.2 vs. 14.7 months; hazard ratio: 0.78, 95% CI: 0.64 to 0.96) [94]. Further analysis showed that ABCP group had an obvious advantage in PFS over BCP group regardless of PD-L1 expression and effector T cell status [94]. Given that first line atezolizumab treatment is limited to non-small-cell lung cancer patients with high PD-L1 expression, the results of IMpower150 are meaningful to expand the application of ICI [95].

### Anti-PD-L1 combined with anti-angiogenesis TKI

In most clinical studies by far, combination strategies consist of ICI and anti-angiogenesis mAb bevacizumab. In 2018 Choueiri et al. firstly reported the efficacy of avelumab plus anti-angiogenesis TKI axitinib therapy in treatment-naïve advanced clear-cell renal-cell carcinoma (JAVELIN Renal 100). JAVELIN Renal 100 (NCT02493751) is a phase 1b study aiming to evaluate safety, pharmacokinetics, and pharmacodynamics of avelumab (anti-PD-L1) plus axitinib (VEGFR TKI) therapy [96]. For a total of 55 patients enrolled in the study, 54 patients received avelumab plus axitinib therapy except for one patient due to abnormally increased blood creatine phosphokinase [96]. Within a follow-up period of nearly one year, 58% (32 of 55) patients showed complete response or partial response to combination therapy while 20% (11 of 55) patients had stable disease [96]. Notably, it was observed that PD-L1 expression did not significantly affect treatment efficacy. Whether choosing cut-off value as 1% or 5%, ORRs of PD-L1 high expression group and PD-L1 low expression group are comparable (cutoff value as 1%: OR 3.80, 95%CI 0.70–18.12; cutoff value as 5%: OR 2.11, 95%CI 0.60–7.57) [96]. Motivated by the encouraging and preliminary results of NCT02493751, a phase 3 clinical trial JAVELIN Renal 101 (NCT02684006) is ongoing to compare the efficacy of avelumab plus axitinib vs. sunitinib monotherapy in advanced clear-cell renal-cell carcinoma.

Later, Xu et al. reported the results of another phase 1 clinical study (NCT02942329) which aimed to investigate the efficacy of SHR-1210 (anti-PD-1 antibody) plus apatinib (VEGFR2 TKI) in refractory hepatocellular cancer (HCC), gastric cancer (GC), and esophagogastric junction cancer (EGJC) patients [97]. 15 patients were assigned to dose escalation group and 28 patients were assigned to dose expansion group (recommended phase II dose of apatinib: 250 mg/d) [97]. Though the efficacy of combination therapy in GC/EGJC patients was unsatisfactory (ORR in evaluable GC/EGJC: 17.4%), the treatment effect in HCC patients was encouraging (ORR in evaluable HCC patients: 50%, 95%CI 24.7–75.4%; disease control rate in evaluable HCC patients: 93.8%, 95%CI 69.8–99.8%; 6-month PFS rate: 51.3%, 95%CI 21.4–74.9%; 9-month PFS rate: 41.0%, 95%CI 13.8 to 66.9%) [97]. Compared with the previous data of nivolumab or VEGFR2 TKI monotherapy, patients gained more benefits from combination therapy [98, 99]. It was presumed that the difference in efficacy among three types of cancers could be attributed to tumor immunogenicity [97]. HCC tends to possess higher immunogenicity than GC and EGJC [97].

### Combination therapy-related adverse event

For ICI therapy, an important factor contributes to treatment discontinuation is the severe adverse event. Most adverse events are related with hyperactive immune response, showing T cell mediated auto-immune like inflammation [100]. Disturbed immune homeostasis results in immune-related damage in normal tissues such as gastrointestinal, skin, and hepatic system [100]. Generally, the risk of anti-PD-1/PD-L1 mAb induced adverse event is lower than anti-CTLA-4 mAb (grade 3–4 adverse event: 7–12% vs. 10–18%) [100]. These adverse events could be alleviated by discontinuing ICI treatment or reducing dose of ICI [64]. Theoretically, anti-angiogenesis promotes tumor vessel normalization, which is favorable to T cell infiltration and drug delivery to tumor. In the combination therapy, we speculated that lower dose of ICI would be sufficient to counteract immunosuppressive microenvironment with less adverse event [64].

## Conclusion

A series of preclinical and clinical studies indicated the mutually enhanced effect of anti-angiogenesis and ICI therapy. On the one hand, anti-angiogenesis blocks the negative immune signals by increasing ratio of anti−/pro-tumor immune cell and decreasing multiple immune checkpoints expression. On the other hand, ICI therapy could restore immune-supportive microenvironment and promote vessel normalization. Besides, because of enhanced drug delivery benefiting from vessel normalization, smaller dose of ICI could be applied which reduces the risk of adverse event. A main problem needing to resolve is how to optimize the dose and schedule of anti-angiogenesis in the combination therapy. Extending window of vessel normalization and avoiding excessive vessel pruning would facilitate the maximized survival benefit. We believe ICI plus anti-angiogenesis would be a promising strategy to overcome treatment resistance and improve patients’ prognosis.

## Abbreviations

ANGPT-2: Angiopoietin-2
bFGF: Basic fibroblast growth factor
CAR: Chimeric antigen receptor
c-FLIP: Cellular FLICE-inhibitory protein
CTLA-4: Cytotoxic T-lymphocyte antigen-4;
DC: Dendritic cell
EC: Endothelial cell
EGJC: Esophagogastric junction cancer
FasL: Fas ligand
Gal-1: Galectin-1
GC: Gastric cancer
HCC: Hepatocellular cancer
HEV: High endothelial venule
IDO: Indoleamine 2, 3-dioxygenase
IFN-γ: Interferon-γ
IL-10: Interleukin-10
IL-6: Interleukin-6
mAb: Monoclonal antibody
MDSC: Myeloid-derived suppressor cell
MHC-I: Major histocompatibility complex-I
MMP: Matrix metalloproteinase
ORR: Objective response rate
OS: Overall survival
PD-1: Programmed cell death protein 1
PFS: Progression-free survival
SCLC: Small-cell lung cancer
TAM: Tumor-associated macrophage
TCR: T cell receptor
TIL: Tumor infiltrating lymphocyte
TKI: Tyrosine kinase inhibitor
Treg: Regulatory T cell
VCAM-1: Vasculature cell adhesion molecule-1
VEGF: Vascular endothelial growth factor
vWF: Von Willebrand factor
